# Impact of the COVID-19 Pandemic on the General Mental Health in Sweden: No Observed Changes in the Dispensed Amount of Common Psychotropic Medications in the Region of Scania

**DOI:** 10.3389/fpsyt.2021.731297

**Published:** 2021-12-01

**Authors:** Mirjam Wolfschlag, Cécile Grudet, Anders Håkansson

**Affiliations:** ^1^Department of Clinical Sciences Lund, Psychiatry, Faculty of Medicine, Lund University, Lund, Sweden; ^2^Department of Psychiatry Malmö-Trelleborg, Malmö Addiction Center, Region Skåne, Kristianstad, Sweden

**Keywords:** COVID-19, public mental health, prescription trends, psychotropic medication, antidepressants, benzodiazepines, anxiety, interrupted time series analysis

## Abstract

Some first investigations have focused on the consequences of the COVID-19 pandemic for the general mental health after its outbreak in 2020. According to multiple self-reporting surveys, symptoms of stress, anxiety, and depression have risen worldwide. Even some studies based on health care records start to be published, providing more objective and statistically reliable results. Additionally, concerns have been raised, to what extend the access to mental health care has been compromised by the COVID-19 outbreak. The aim of this study was to detect changes in prescription trends of common psychotropic medications in the Swedish region of Scania. The monthly dispensed amounts of selected pharmaceuticals were compared from January 2018 until January 2021, regarding the prescription trends before and after the outbreak of COVID-19. Using an interrupted time series analysis for each medication, no general trend changes were observed. On the one hand, a possible deterioration of the general mental health could not be confirmed by these results. On the other hand, the access to mental health care did not seem to be impaired by the pandemic. When interpreting findings related to the COVID-19 pandemic, regional differences and country-specific approaches for coping with the pandemic should be considered. The Swedish population, for instance, never experienced a full “lock-down” and within Sweden the time point of the outbreak waves differed regionally. In general, the effects of the COVID-19 outbreak on mental health are still unclear and need to be investigated further in an international comparison.

## Introduction

Since the outbreak of the COVID-19 pandemic in spring 2020, it is generally assumed that public mental health was affected negatively by the worldwide situation (1). Multiple factors related to the crisis could possibly be influencing mental health, both direct restrictions to everyday life but also the unpredictable situation worldwide and the necessity to accept disease and death as more present topics than earlier (2). Within the last year, some first attempts have been made to assess the mental health state of the general population. The most common research tool to gain a basic impression of the situation so far have been self-reporting surveys (3). They have been conducted in many different countries and have often been screening for increased symptoms of stress, anxiety, and depression, see for instance (4–9). In most cases, a moderate to severe impact by the pandemic on the mental health was reported by a notable proportion of the participants. While being an applicable method to create an overview over the subjective experiences within the pandemic, there are limitations to surveys based on self-evaluation (3). The selection process for the participant samples is often biassed toward certain groups within the population. Accessibility to the internet can be an issue, possibly excluding persons of high age, severe illnesses, or an underprivileged social status. Furthermore, self-reported symptoms are less reliable compared to a diagnosis by a professional. Therefore, research based on health care records may be a more suitable method for yielding statistically valid results and an important resource for decision-making.

To date, very few findings based on information from the health care systems have been published regarding the general mental health during the COVID-19 pandemic. Carr et al. explored the consequences of the pandemic for mental health using the primary care records of over 14 million patients in the UK (10). Different parameters were examined to determine the developments in mental health such as the incidence of depression and anxiety diagnoses, the number of prescriptions for antidepressants and benzodiazepines, the referral to mental health services, and reported self-harm episodes. All these parameters declined remarkably after March 2020 with the onset of the COVID-19 pandemic and increased again to their normally expected levels in September 2020. Since such a sudden, strong, and temporary amelioration of the general state of mental health seems unlikely and contradicts the results of a large survey study in the UK (11), a compromised access to mental health care could be a more probable explanation for the findings. Similar concerns were expressed by the authors of a study in the U.S., where the increased potential need for mental health services was estimated much higher than the increase in treatment-seeking during the pandemic (12). Another reason for the divergence between needing help and seeking help could be the fear within the population to be exposed to a higher infection risk when being in contact with medical facilities. In Germany, a database study showed increased numbers of newly diagnosed anxiety disorders (+21%) between March and June 2020 compared to the same months in 2019, using mental health care records of 1.9 million patients (13). However, within these newly diagnosed patients, the prescriptions for antidepressants, benzodiazepines, and herbal sedatives were reduced in comparison to earlier diagnosed patients.

Research on the Swedish population could contribute to a better understanding of the relation between the COVID-19 outbreak and alterations in mental health, since the measures taken against the pandemic in Sweden differ strongly from the ones applied in most countries. A real “lockdown” was avoided and the strategy of relying on recommendations, rather than restrictions, lead to an everyday life differing less from the conditions before the pandemic than in most parts of the world (14, 15). The results of a self-reporting survey in Sweden were in line with the worldwide trend of increased depression and anxiety symptoms and the demand for help via the Swedish suicide hotline increased strongly after the outbreak of the pandemic (16, 17). Additionally, the National Board of Health and Welfare performed a nationwide analysis of the mental health state under the pandemic (18). Regarding psychotropic medication, a decrease in new prescriptions for antidepressants and an increase in new prescriptions within attention-deficit/hyperactivity disorder (ADHD) were noted. Overall treatment-seeking within the mental health care system was unaffected and constant in numbers over the last years, while the number of new psychiatric patients decreased after the COVID-19 outbreak. In line with these findings, another study observed decreased contact to the health care system regarding acute cases of depression and anxiety (19).

The lack of analysed data based on mental health care systems impairs profound conclusions and fact-based decision-making in relation to the COVID-19 pandemic. One indicator for the mental health state and the contact to the health care system within the population are psychotropic prescription numbers. Thus, the aim of our study was to present an overview over the dispensed amounts of common psychotropic medications, compared between January 2018 and January 2021. As mentioned before, the Swedish population faced milder restrictions during the pandemic than most other countries and our results could therefore be especially interesting in the international comparison. We had access to all pharmacy records for the Swedish region of Scania, providing the possibility of studying the prescription numbers for the total population within this area. The goal was to detect any changes in prescription trends that could be related to the COVID-19 outbreak.

## Materials and Methods

This study included all dispensed prescriptions for a large number of selected psychotropic medications in the region of Scania in southern Sweden [1.38 million inhabitants in December 2019 (20)], whether or not these were prescribed in specialised psychiatry, by general practitioners, or by any other health care service in the region. Since the data were obtained from pharmacies as common suppliers, they could not be studied separately regarding their prescription setting. The data contained complete records of the selected medications dispensed between January 2018 and December 2020/January 2021. Thereby, we considered our analysis to be based on a sufficient amount of datapoints before and after the intervention point March 2020, securing statistically reliable results. The overall dispensed amount per month was recorded in defined daily doses (DDDs).

With the goal of getting an overview over the mental health state of the general population, the most commonly prescribed psychotropic drug classes were chosen for the analysis, each of them dispensed in at least 200,000 DDDs per month in Scania over the last 3 years. Additionally, the common tranquilisers and anxiolytics promethazine, alimemazine, and hydroxyzine were included in the analysis, each of them dispensed in at least 100,000 DDDs per month in Scania within the last 3 years. All analysed drug classes and single drugs are listed in Table 1, including anatomical therapeutic chemical (ATC) codes. Sedative benzodiazepines (ATC code N05CD) were excluded from the analysis, since both nitrazepam and flunitrazepam are being taken off the Swedish market and decreased considerably in their dispensed amounts over the last 3 years (21, 22).

**Table 1:** Trends, changes of trends, and respective *P*-values from the ITS analysis of the most common psychotropic medications.

**Name**	**ATC code**	**Trend in DDD/month**	***p*-value (trend)**	**Change of trend in DDD/month**	***p*-value (change of trend)**
Antidepressants	N06A	+23,794 ± 3,101	<0.001	+22,053 ± 14,805	0.146
Benzodiazepine related drugs	N05CF	−543 ± 1,920	0.779	+7,095 ± 8,987	0.436
Psychostimulants, agents used for ADHD (also including guanfacine), and nootropics	N06B and C02AC02	+4,995 ± 490	<0.001	+3,947 ± 2,301	0.096
Other hypnotics and sedatives	N05CM	+2,823 ± 494	<0.001	+3,589 ± 2,351	0.137
Anxiolytic benzodiazepines	N05BA	−1,142 ± 223	<0.001	+803 ± 1,065	0.456
Mood stabilisers (Quetiapine, lamotrigine, and lithium)	N05AH04, N03AX09, N05AN	+1,531 ± 195	<0.001	+345 ± 1,113	0.759
Promethazine	R06AD02	+3,506 ± 323	<0.001	+2,270 ± 1,454	0.128
Alimemazine	R06AD01	+734 ± 187	<0.001	+2,668 ± 868	0.004
Hydroxyzine	N05BB01	+53 ± 170	0.757	+987 ± 771	0.210

The aim was to identify any change in trend for the monthly dispensed amount of each medication. Thus, an interrupted time series (ITS) analysis was performed using the software IBM SPSS Statistics 26. The applied model was a non-seasonal autoregressive integrated moving average (ARIMA) with the main parameter of interest being the change in slope. A periodicity of 12 months was taken under consideration and March 2020 was used as an intervention point, given that the COVID-19 outbreak in Sweden occurred within this month. Any slope, or change of slope, was considered significant for a *p* < 0.05.

As the study did not involve any individual clinical data that can be referred to an identified person, no ethical permission was required.

## Results

**Figure 1:**
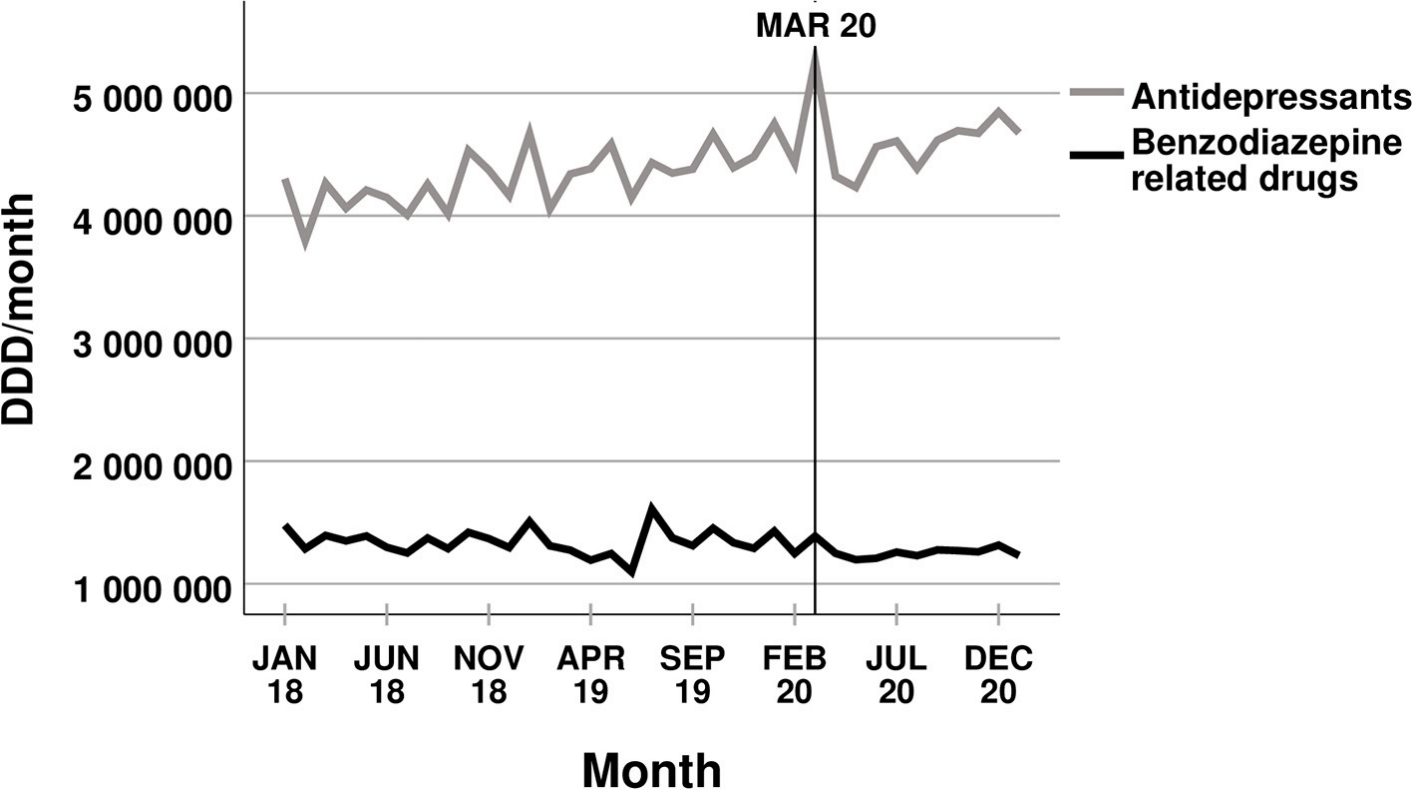
Dispensed amounts of antidepressants and benzodiazepine related drugs.

**Figure 2:**
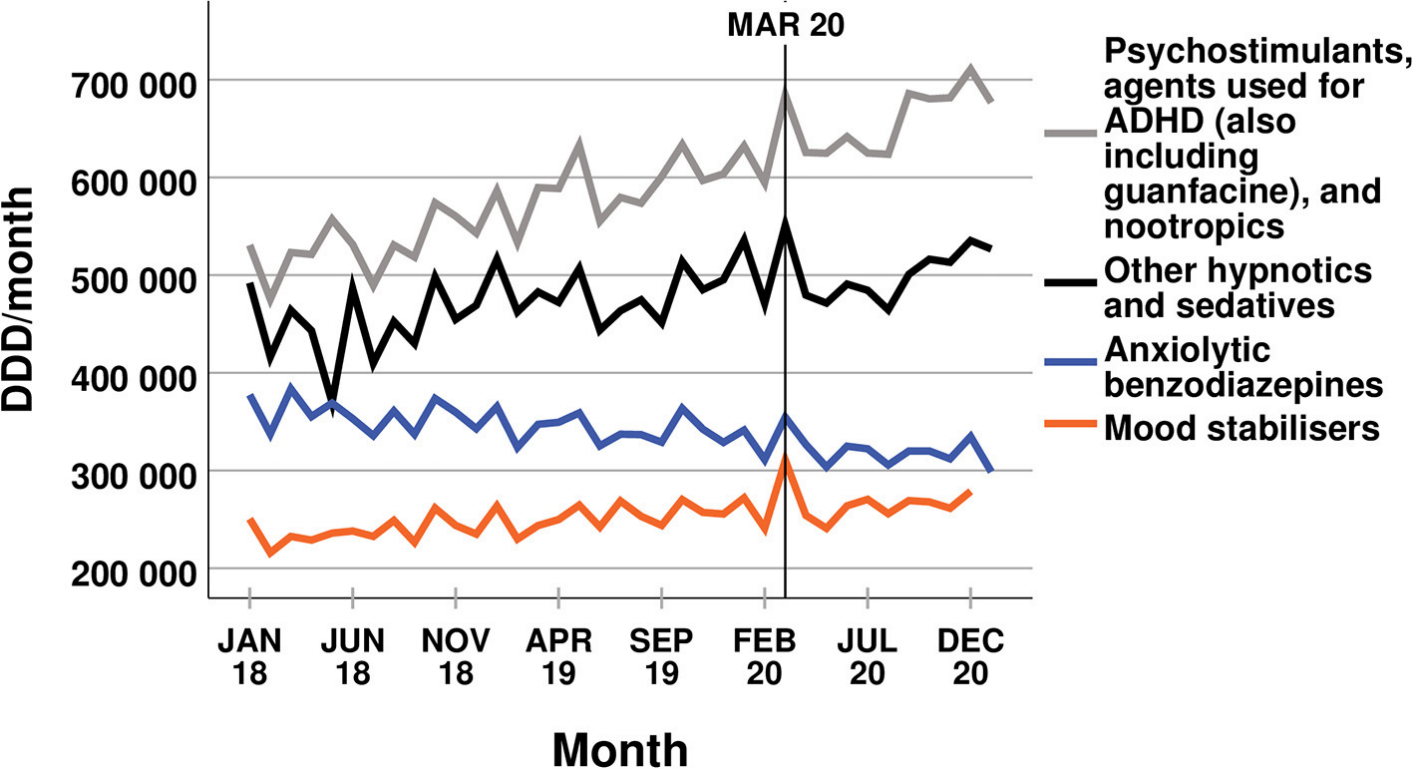
Dispensed amounts of psychostimulants, agents used for ADHD (also including guanfacine), and nootropics, other hypnotics and sedatives, anxiolytic benzodiazepines, and mood stabilisers.

**Figure 3:**
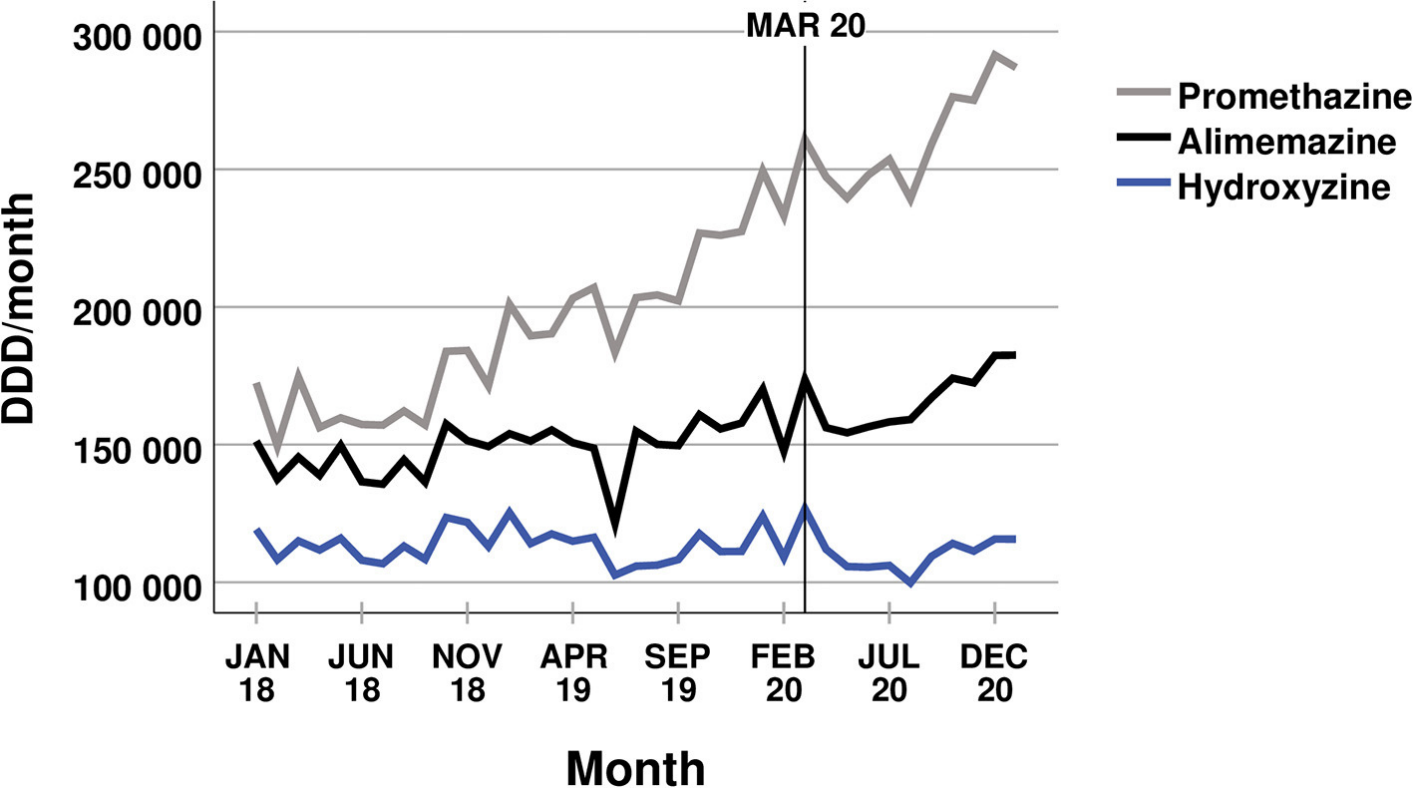
Dispensed amounts of promethazine, alimemazine, and hydroxyzine.

Overall, the trends within the dispensed amounts of common psychotropic medications did not change after the COVID-19 outbreak in Scania. None of the analysed drug classes showed a significant change to the ongoing trend that had occurred before March 2020 (see Figures 1, 2). The dispensed amount of antidepressants, psychostimulants, agents used for ADHD (also including guanfacine), and nootropics, other hypnotics and sedatives, and mood stabilisers continued to increase after the COVID-19 outbreak. The disposal of anxiolytic benzodiazepines kept on decreasing, while there was no significant trend to the dispensed amount of benzodiazepine related drugs before or within the pandemic. Even the disposal of the two drugs promethazine and hydroxyzine showed no significant change in trend related to the COVID-19 pandemic (see Figure 3). The amounts of dispensed promethazine kept on rising and there was no consistent trend in the disposal of hydroxyzine. However, as a single exception, the dispensed amount of alimemazine increased more strongly after the COVID-19 outbreak (see Figure 3) with 2,668 ± 868 DDDs additionally more per month (*p* = 0.004). All trends, changes in trends, and their respective *p*-values are listed in Table 1. Despite the stability in trends before and within the pandemic, almost all dispensed amounts of the different medications show a temporary peak in March 2020.

## Discussion

In general, the prescription trends of common psychotropic medications did not change in the region of Scania after March 2020, with the sedative alimemazine as an exception. Thus, we interpret our results as negative findings regarding changes of prescription trends due to the COVID-19 pandemic. The mental health of the general population does not seem to be affected in a way that results in an increased use of psychotropic medication so far, even if other treatment and support options, such as psychotherapy and group meetings, have become more difficult to conduct during the pandemic (23).

Most likely, the explanation for the recent increase in dispensed amounts of alimemazine is its application as a replacement for other sedative drug classes. Benzodiazepines have been criticised for their adverse effects and antihistamines are used as one possible pharmacological alternative (24). According to our findings, the prescriptions for anxiolytic benzodiazepines have decreased remarkably over the last years. This could also explain the visible, yet not significant, upward trend in the dispensed amounts of promethazine. It can therefore be assumed that the rises in antihistaminic prescriptions are not a consequence of the pandemic.

The temporary spikes in dispensed numbers for almost all prescriptions in March 2020 were probably caused by a general uncertainty in the population at the beginning of the pandemic, which resulted in panic buying of certain goods, including pharmaceuticals. Additionally, certain information about a possible shortage of medication due to the pandemic was spreading in the media in March 2020, which presumably led to many patients taking out their medication in higher amounts than usual as a precaution (25).

We expect the analysed prescription trends for common psychotropic medication to be a reliable indicator for more severe mental health problems in the general population, which seem unchanged by the pandemic. Furthermore, our findings could support the conclusion, that the access to mental health care in Scania has not been compromised during the pandemic. Since the prescription trends were not affected by the COVID-19 outbreak, patients seem to have received the help they needed within this area of treatment. On the other hand, a negatively affected public mental health state and impaired possibilities to seek help could potentially compensate each other in the recorded dispensed amounts of psychotropic medication and hence become undetectable by this study design. As mentioned in the introduction, several studies in Sweden suggest a deterioration in mental health and a compromised access to mental health care (16–19). Especially new and acute patients seem to be affected, perhaps struggling the most with searching help while avoiding physical contact during the pandemic. Since this study included all psychiatric patients, it might not be sensitive to this effect and could indicate that patients under long-term treatment found a way to maintain their contact to the health care system.

Two factors should be considered when comparing these results to other studies. First, as mentioned in the introduction, Sweden chose a different epidemiological approach to the pandemic than most countries. On the one hand, this could have contributed to a less stressful experience for some individuals by protecting normal routines and possibilities. On the other hand, certain individuals, perhaps especially vulnerable to the pandemic, might have perceived the actions in Sweden as insufficient, resulting in increased stress. Even if Sweden is characterised by special circumstances during the pandemic compared to the rest of the world, our results are still valuable on an international level. The daily life might have been impacted in different ways in Sweden, but the Swedish population was equally exposed to the extreme changes worldwide, such as anxiety about the future or travel bans. Transferring our study design to other countries could create a comparability of the public mental health state in different countries during the pandemic that no other design could provide so far.

The second factor possibly influencing the results of this study, are the special circumstances in the region of Scania during the pandemic. While most regions in Sweden were affected by the first wave of rapidly rising infection rates, starting in March 2020, the spread in Scania was much less severe during this time. Instead, the first wave in Scania occurred in autumn 2020 rather than in spring 2020 (26, 27). Taking this fact into account, we additionally performed an ARIMA analysis with October 2020 as an intervention point, yielding similar results to the first analysis. Given that the second analysis was based on no more than three, respectively, two, datapoints after the intervention point, we decided to consider it as statistically unreliable and focused on March 2020 as an intervention point instead. Since the outbreak worldwide and in Sweden in general can clearly be dated to the original intervention point, we believe our analysis to be valid, even if the impact of the local situation might not be taken under consideration with this choice of methods. We are aware that the regional deviations in Scania compared to the rest of Sweden might influence the results and plan to perform a new analysis with October 2020 as an intervention point as soon as we have access to more recent data from 2021.

The impact of the COVID-19 pandemic on mental health is a multifactorial and complex issue. To date, most conclusions are based on speculations rather than reliable data and much more research is needed to create a profound overview over the situation worldwide. General prescription trends are a suitable method to obtain a broad impression over the public mental health state. An additional study design could focus separately on prescriptions and diagnoses given for the first time during the pandemic and patients treated already before the pandemic. Thereby, the effect of the pandemic on previously healthy subjects and patients already in a vulnerable mental health state could be investigated more distinctly. Furthermore, survey studies performed within the psychiatric care system could provide more reliable and less biassed results than the ones obtained by questionnaires sent out to the general public. This approach could yield more detailed information on the actual personal situations during the pandemic and highlight possible causes for a negatively impacted mental health. However, data collected by addressing patients individually would be less suitable for examining broader trends or creating an overview over the general situation.

On a final note, even without confirmed knowledge about a possibly impaired access to mental health care, the health care systems should be prepared for a considerable increase in new psychiatric patients in the aftermath of this pandemic. Many preliminary studies indicate a broad and detrimental effect on mental health by the recent situation, though their results are not sufficient for evidence-based conclusions yet (28).

## Data Availability Statement

The dataset will be available from the authors upon request. Requests to access these datasets should be directed to mirjam.wolfschlag@kabelmail.de.

## Author Contributions

CG and AH were responsible for concept and design of the study. AH obtained the data. MW performed the statistical analysis and wrote the manuscript. All authors contributed to manuscript revision, read, and approved the submitted version.

## Conflict of Interest

AH holds a position at Lund University which is supported by the state-owned Swedish gambling operator AB Svenska Spel as part of its responsible gambling policies, and also has research funding from the research councils of that body and from the research council of the Swedish alcohol monopoly, Systembolaget. AH also has been the national sub-investigator of a pharmaco-epidemiological survey, carried out by the independent research institute Triangle Research Institute, United States, and financed by the pharmaceutical company Shire. No direct fees or compensations were paid to the individual sub-investigator. Also, a clinical follow-up study has been planned in collaboration with the company Kontigo care, which provides follow-up devices free of charge for a clinical treatment trial in gambling disorder, but without any further financial support to the research group or its members. None of the bodies mentioned above had any role in or influence on the present research. The remaining authors declare that the research was conducted in the absence of any commercial or financial relationships that could be construed as a potential conflict of interest.

## Publisher's Note

All claims expressed in this article are solely those of the authors and do not necessarily represent those of their affiliated organizations, or those of the publisher, the editors and the reviewers. Any product that may be evaluated in this article, or claim that may be made by its manufacturer, is not guaranteed or endorsed by the publisher.
